# Supplementary material for: Solving complex nanostructures with ptychographic atomic electron tomography
Source: Nat Commun. 2023 Nov 30;14:7906. doi: 10.1038/s41467-023-43634-z (PMC10689721; doi:10.1038/s41467-023-43634-z)
Supplement: Supplementary file 1 — Supplementary Information [file 41467_2023_43634_MOESM1_ESM.pdf]

Supplementary Material for:
Solving Complex Nanostructures With Ptychographic Atomic Electron Tomography

Philipp M. Pelz,[1,2,3,*] Sinéad Griffin,[3,4] Scott Stonemeyer,[4,5,6,7] Derek Popple,[4,5,6,7] Hannah Devyldere,[2] Peter Ercius,[3] Alex Zettl,[2,4,5,7] Mary C. Scott,[2,3] and Colin Ophus[3,†]

[1] *Institute of Micro- and Nanostructure Research (IMN) & Center for Nanoanalysis and Electron Microscopy (CENEM), Friedrich Alexander-Universität Erlangen-Nürnberg, IZNF, 91058 Erlangen, Germany*
[2] *Department of Materials Science and Engineering, University of California Berkeley, Berkeley, CA 94720*
[3] *The Molecular Foundry, Lawrence Berkeley National Laboratory, Berkeley, California 94720, United States*
[4] *Materials Sciences Division, Lawrence Berkeley National Laboratory, Berkeley, California 94720, United States*
[5] *Kavli Energy NanoSciences Institute at the University of California at Berkeley, Berkeley, California 94720, United States*
[6] *Department of Chemistry, University of California at Berkeley, Berkeley, California 94720, United States*
[7] *Department of Physics, University of California at Berkeley, Berkeley, California 94720, United States*

EXPERIMENTAL PARAMETERS FOR TEAM 0.5

Microscope Voltage	80 keV
Electron gun	S-FEG
Source size (FWHM)	0.8 Å
Cc	0.6 mm
Defocus spread (FWHM)	6 nm
Convergence semi-angle	25 mrad
Depth of field	9 nm
Detector	4D Camera @ 87 kHz
Detector pixel size	4.14×10^{-3} Å^{-1} / 173.6 µrad
Detector pixel size (binned)	0.0272 Å^{-1} / 1.136 mrad
Detector outer angle	50 mrad
Reconstruction pixel size	0.397 Å
Energy filter	No
Number of projections	36
Tilt range	−53.9 deg
	65.2 deg
Total recorded diffraction patterns	34 560 000
Manually selected diffraction patterns	8 423 259
STEM step size	0.397 Å
STEM dwell time	11.49 µs
Probe current	40 pA
Electron fluence accumulated	$6.28 \times 10^5\ e/$Å^2
Electron fluence per projection	$1.72 \times 10^4\ e/$Å^2
Avg. electrons per diffraction pattern	2726

Table I. Experimental parameters for PAET.

COMPARISON OF IMAGING MODES

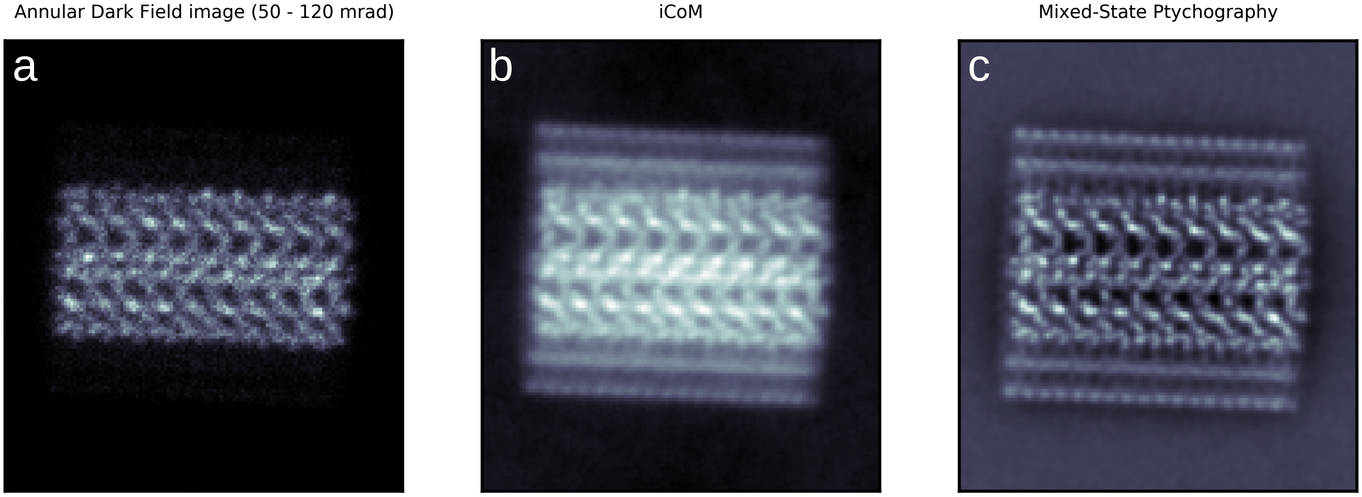

Figure 1. Comparison of a) ADF-STEM, b) iCoM, and c) mixed-state ptychography imaging modes of the nanowire simulated with abtem at the experimental electron fluence of $1.72 \times 10^4\ e/\text{Å}^2$. ADF and iCoM signal were simulated with an aberration-free probe, while mixed-state ptychography was simulated and reconstructed with the experimental probe aberrations.

FULL FIELD OF VIEW RECONSTRUCTION

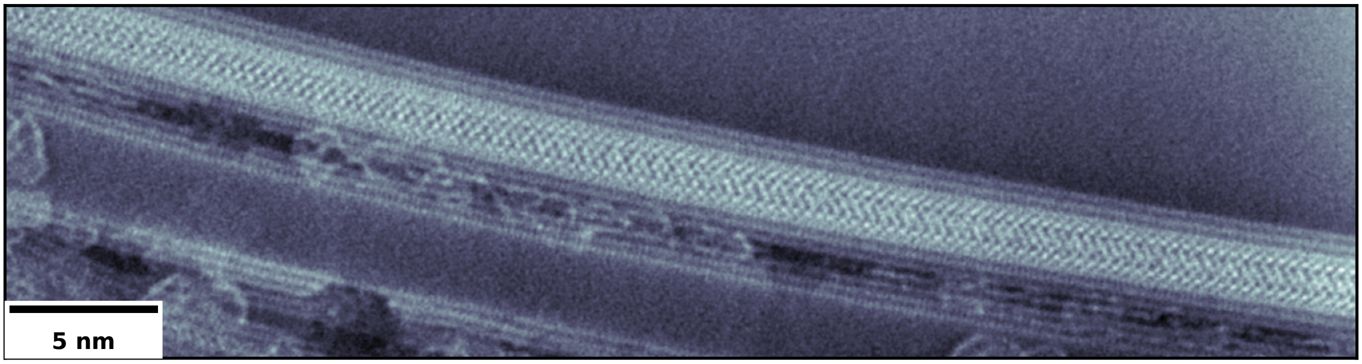

Figure 2. Mixed-state ptychography reconstruction of the full field of view from 1600 x 600 diffraction patterns at 12.7 degree tilt. A triple-wall carbon nanotube (TW-CNT) is directly adjacent to the reconstructed nanotube. At high tilt angles, the TW-CNT partially overlaps with the filled DW-CNT.

PTYCHOGRAPHIC TILT SERIES, NO UNIT-CELL AVERAGING

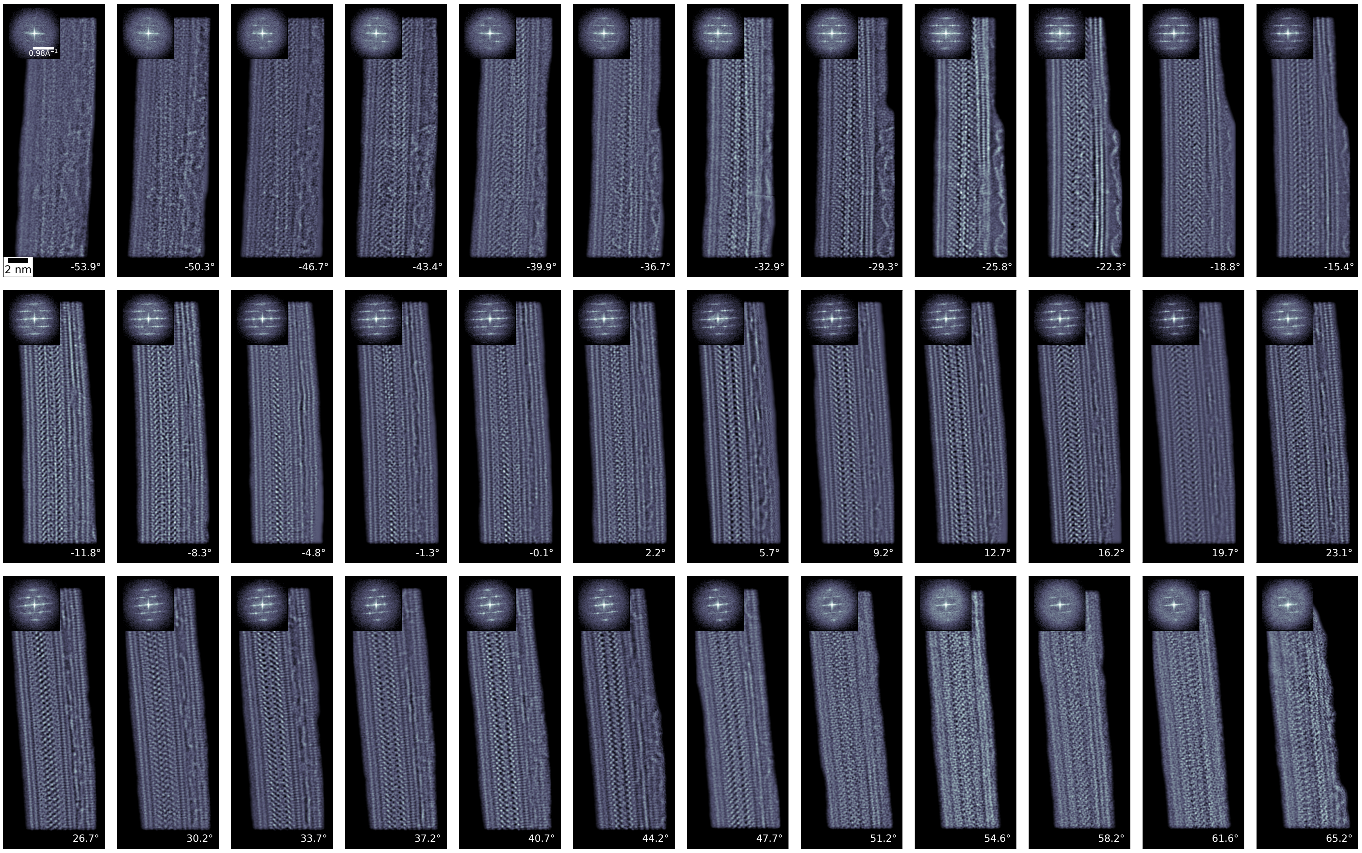

Figure 3. Ptychographic electron tomography tilt series of the the multi-walled carbon nanotube filled with $ZrTe_x$. The 36 phase-contrast projection images with a tilt range from -53.9 to 65.2 degrees (shown at bottom right of each panel) were measured with the 4D Camera and reconstructed using the LSQML algorithm. The total electron dose of the experiment is $6.28 \times 10^5\, e/\text{Å}^2$. The inset shows the power spectrum of each projection.

PTYCHOGRAPHIC TILT SERIES OF HIGH-QUALITY UNIT-CELL AVERAGES

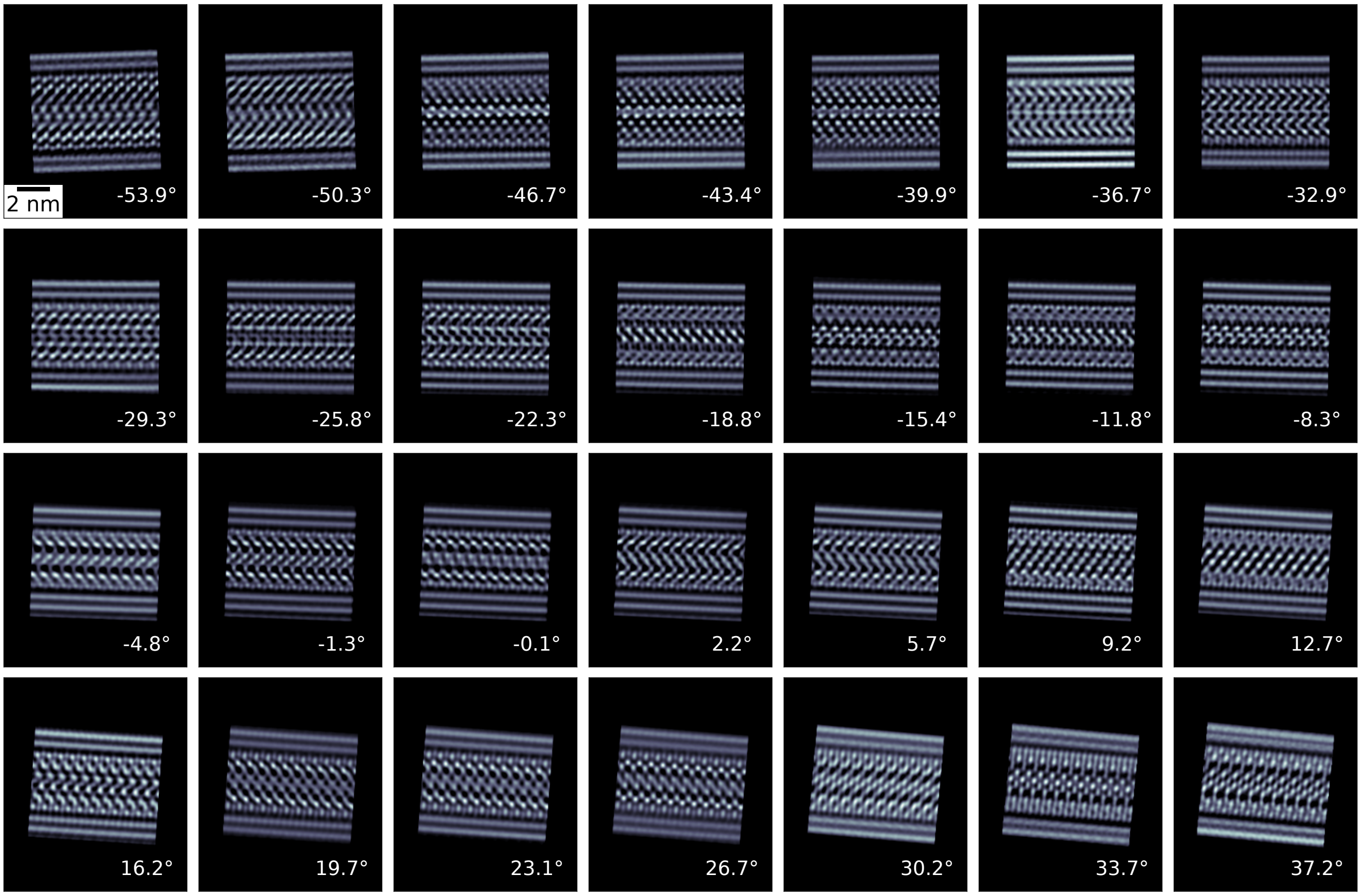

Figure 4. Tilt series of unit-cell averaged ptychographic phase-contrast images from −53.9° to 37.2°.

PTYCHOGRAPHIC RECONSTRUCTION PARAMETERS

Nprobes	4
Nmodes	4
method	MLs
Niter	115
probe_pos_search	10
beta_object	1
beta_probe	1
likelihood	amplitude
grouping	200
apply_subpix_shift	true
variable_probe	true
variable_probe_modes	1
variable_intensity	false
beta_LSQ	true
apply_multimodal_update	false
delta_p	0.1
probe_inertia	0.1
W	0
object initialization	constant phase
probe initialization	defocus from interactive single-sideband reconstruction [1]

Table II. Reconstruction parameters for LSQML reconstruction.

RECONSTRUCTED PROBE WAVE FUNCTIONS

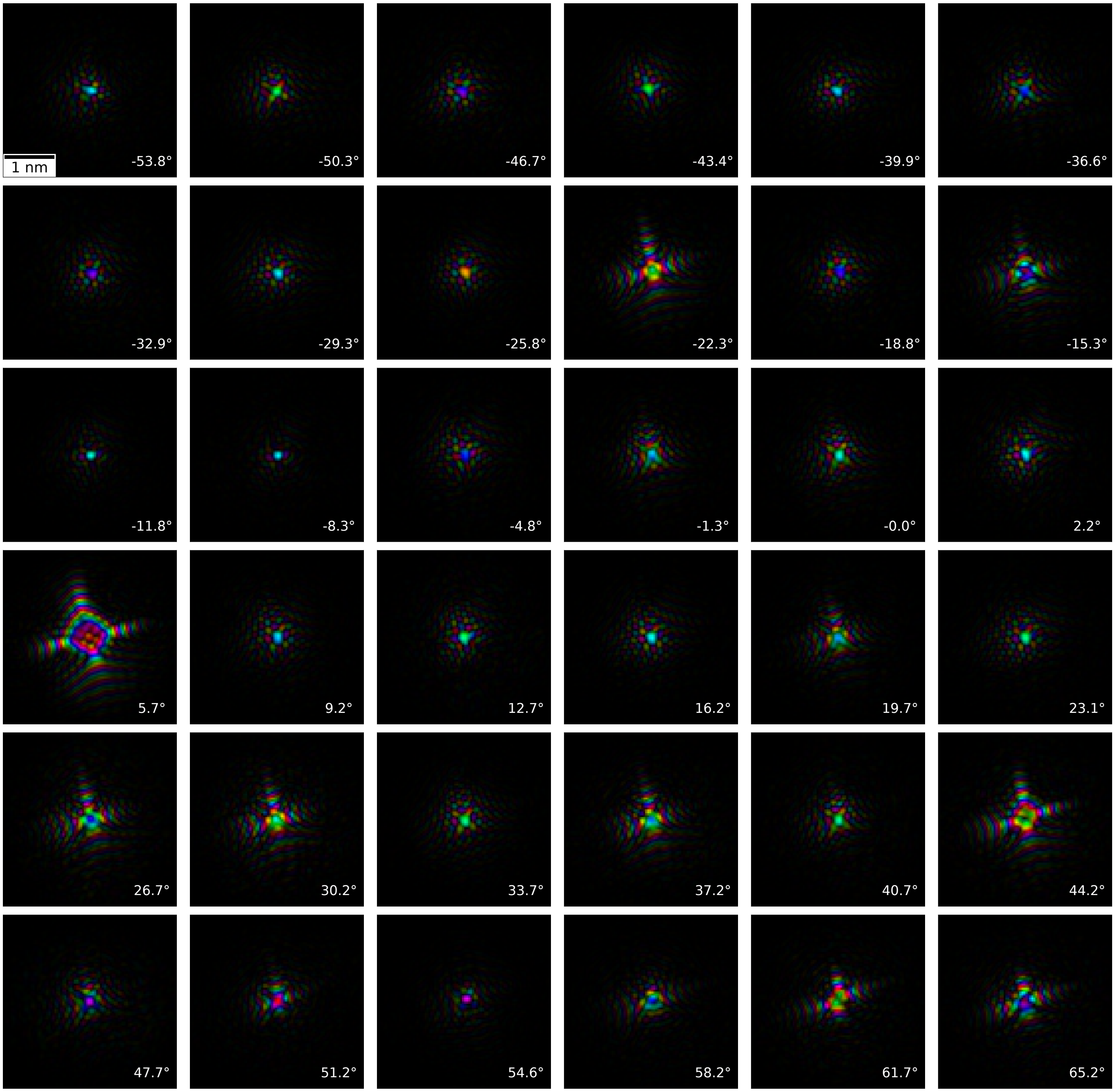

Figure 5. Reconstructed probe wave function for each tilt. Amplitude is shown using color saturation, while phase of each pixel is given by the hue.

COMPARISON OF LINEAR MODEL AND MIXED-STATE PTYCHOGRAPHIC INVERSION FROM FULL MULTI-SLICE SIMULATION

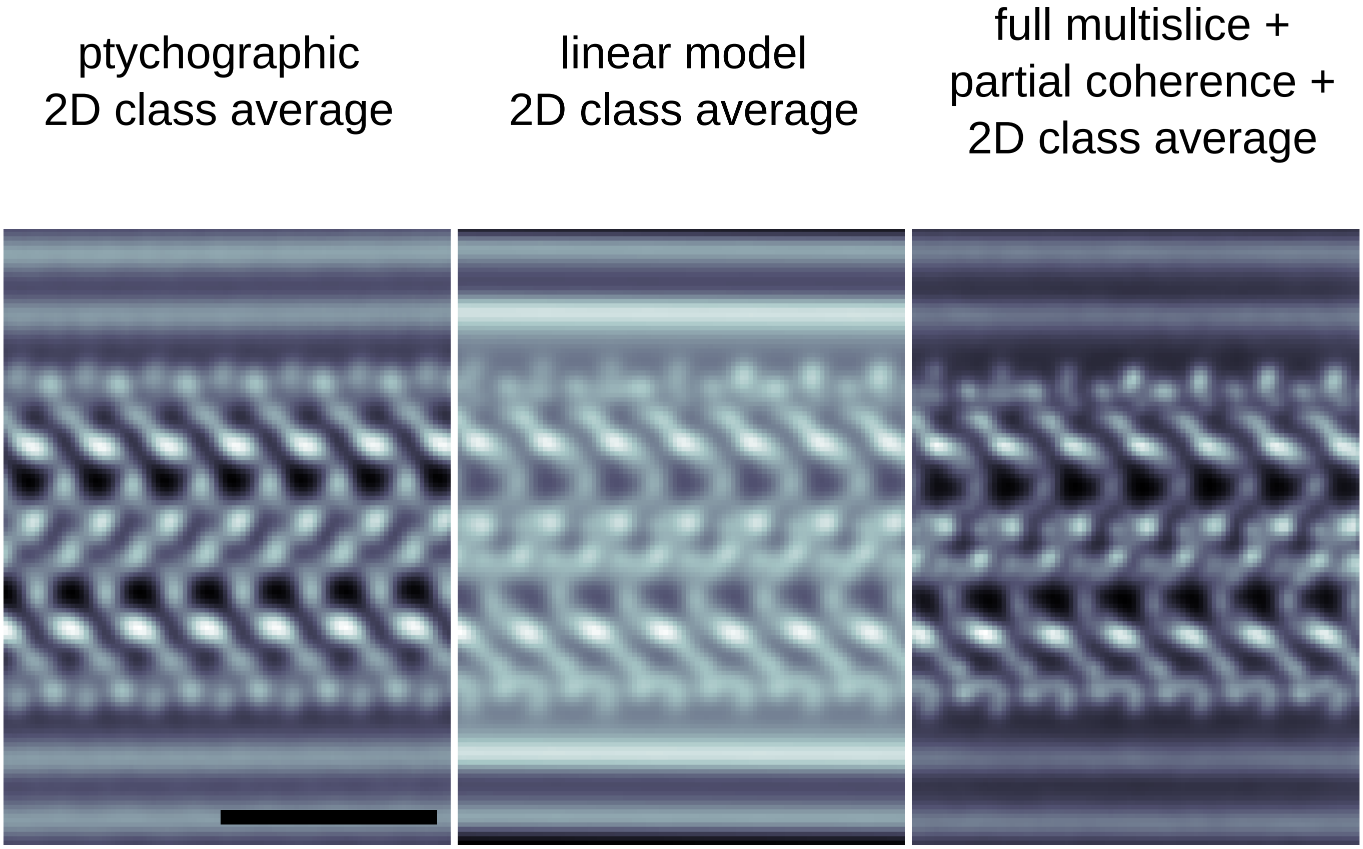

Figure 6. Left panel: 2D class average obtained from experimental mixed-state ptychography reconstruction. Middle: 2D unit-cell average obtained from 12 atomic models where the carbon nanotube was shifted by multiples of 17.75 pm along the tube direction. Right Panel: 2D unit-cell average obtained from 12 ptychographic reconstructions of 4D-STEM datasets simulated with the PRISM algorithm and partial spatial and temporal coherence effects accommodated. The carbon nanotube was shifted by multiples of 17.75 pm along the tube direction. Scale bar: 2 nm

RECONSTRUCTIONS BEFORE, DURING AND AFTER THE SCAN

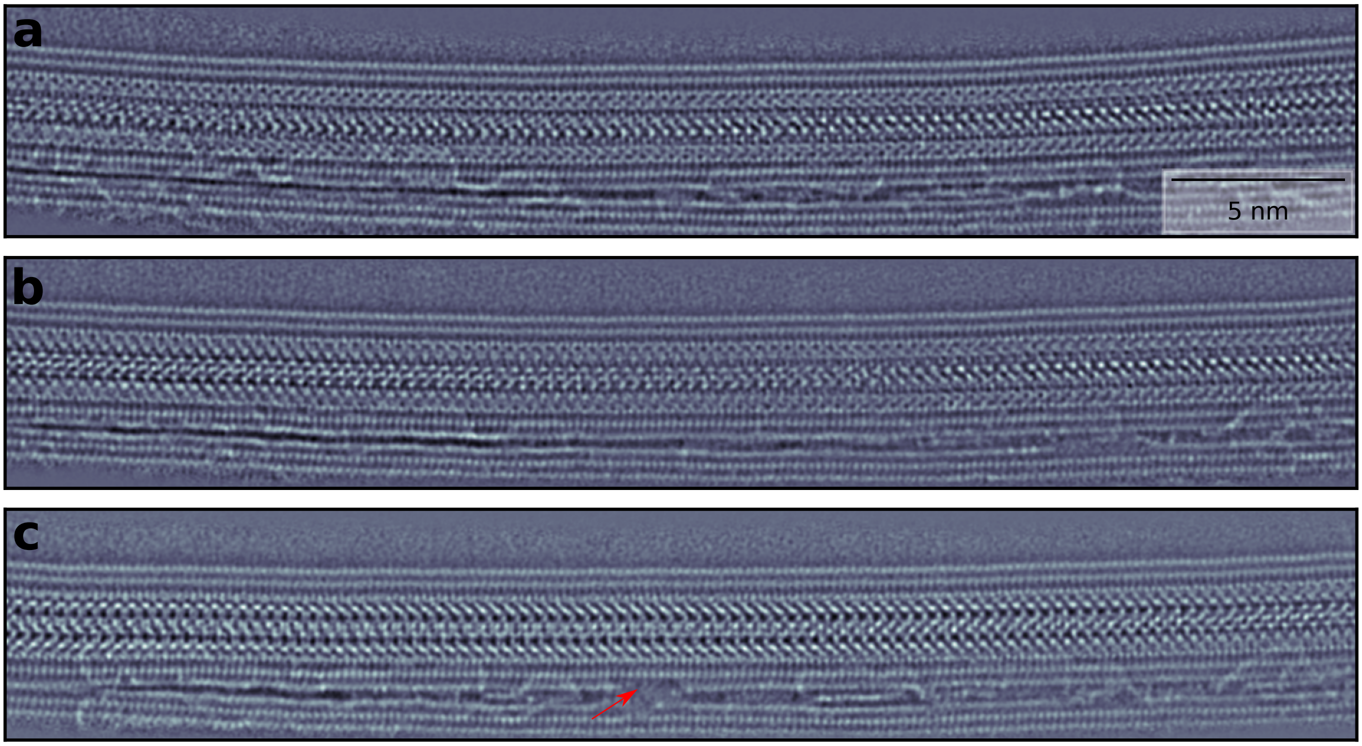

Figure 7. Reconstructed projections a) before, b) during and c) after the tilt series, close to 0° tilt. c) has a slightly different viewing angle due to tilt hysteresis. Only minor damage to the DW-CNT is visible after the tilt series is taken, indicated by the red arrow in c).

PRECISION OF THE ATOMIC COORDINATES

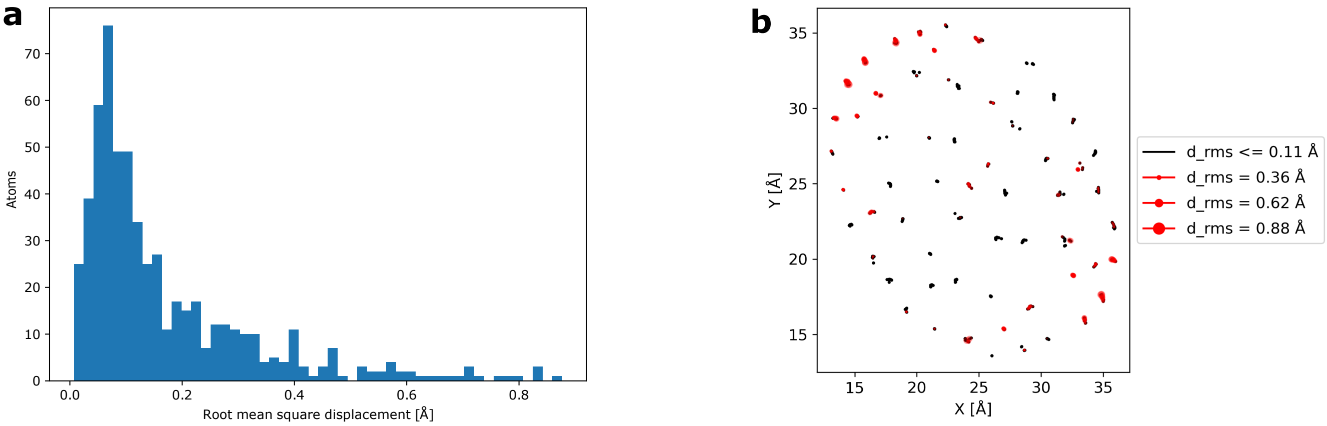

Figure 8. a) Histogram of the distance between experimentally determined coordinates and coordinates obtained from traced coordinates from a simulated tilt series with the determined atomic model. Mean position error is 17 pm and median position error is 10 pm. b) Spatial distribution of the position errors of the Zr & Te atoms, viewed along the nanotube.

CHEMICAL CLASSIFICATION BY NEAREST NEIGHBORS

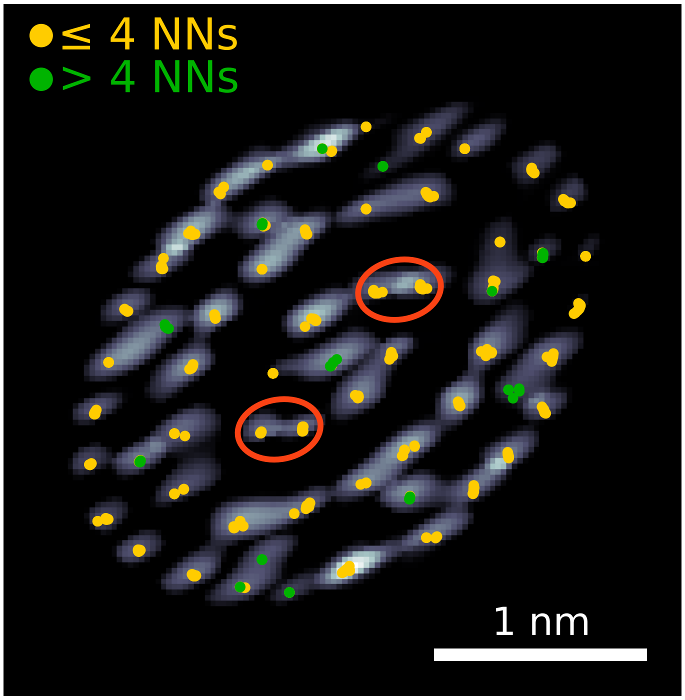

Figure 9. Projection of the core of the nanotube along the nanotube axis. Overlayed are the color-coded traced atomic positions depending on the number of the nearest neighbors (NNs). Atomic columns with predominantly less or equal 4 NNs were assigned as Te, while columns with predominantly greather than 4 NNs were asssigned Zr. The chemical species of the circled atoms was determined by choosing the lowest-energy configuration as determined by density functional theory.

FULL VOLUME RECONSTRUCTION, DEPTH OF FOCUS

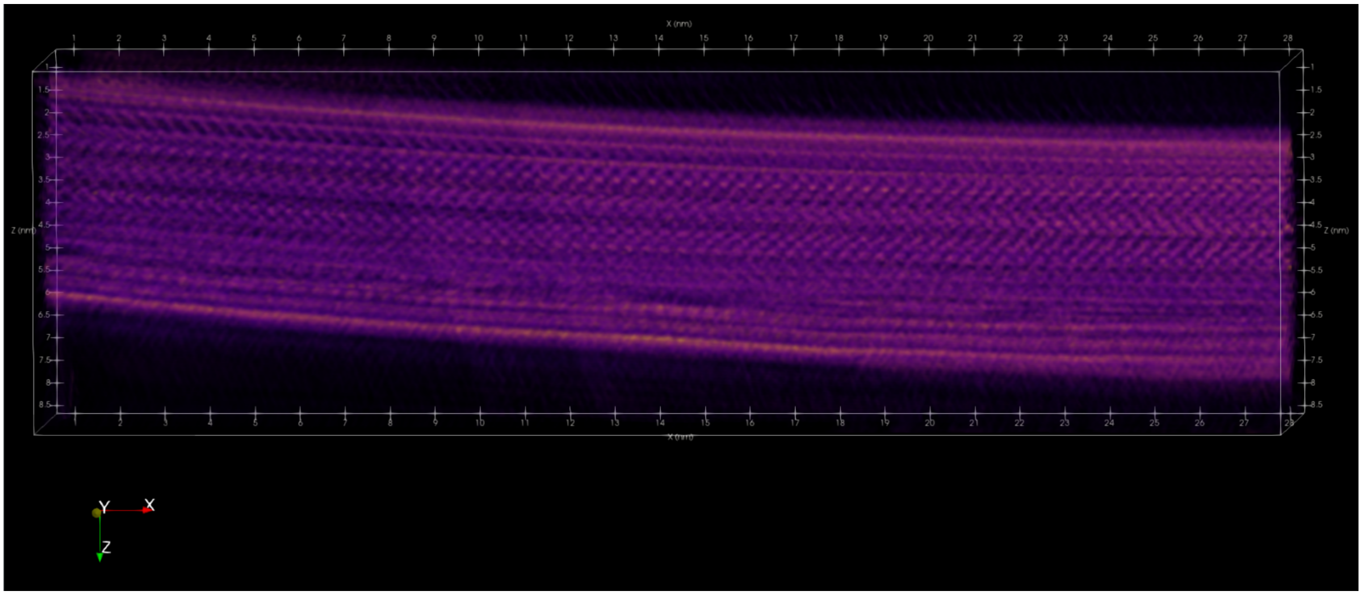

Figure 10. Full reconstructed volume. The z-position of the center of the DW-CNT changes by approx. 2 nm from the left to the right of the volume. The total thickness is smaller than the depth of field of 9 nm.

DATASET CHUNKING SCHEME

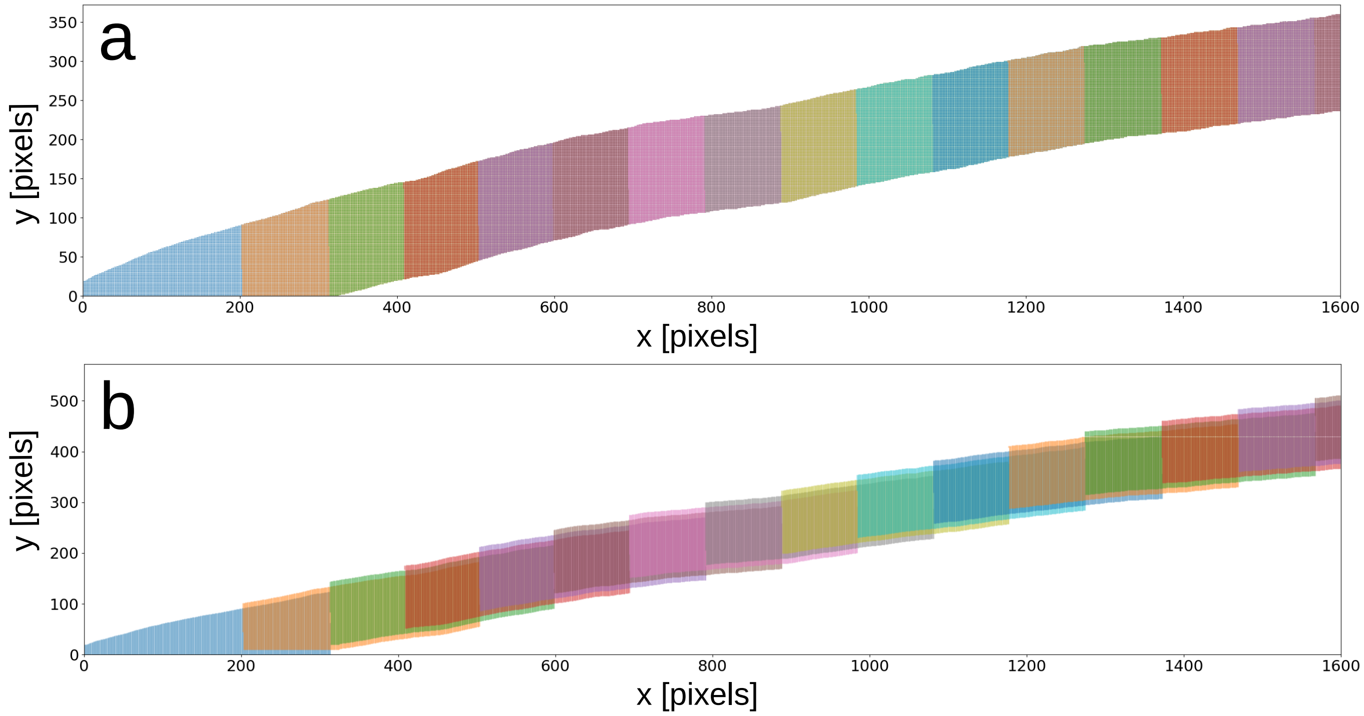

Figure 11. Chunking scheme used for ptychographic reconstruction. Each dataset was divided into overlapping chunks of 36000 diffraction patterns. a) scatterplot of the scan position chunks at tilt angle 5.69° b) the same scatterplot, but the positions of each chunk are shifted vertically, such that the overlap between chunks is clearer. b) is merely for visualization purposes, the input positions are shown in a).

RECONSTRUCTED PROBES IN THE CONDENSER PLANE (FOURIER SPACE)

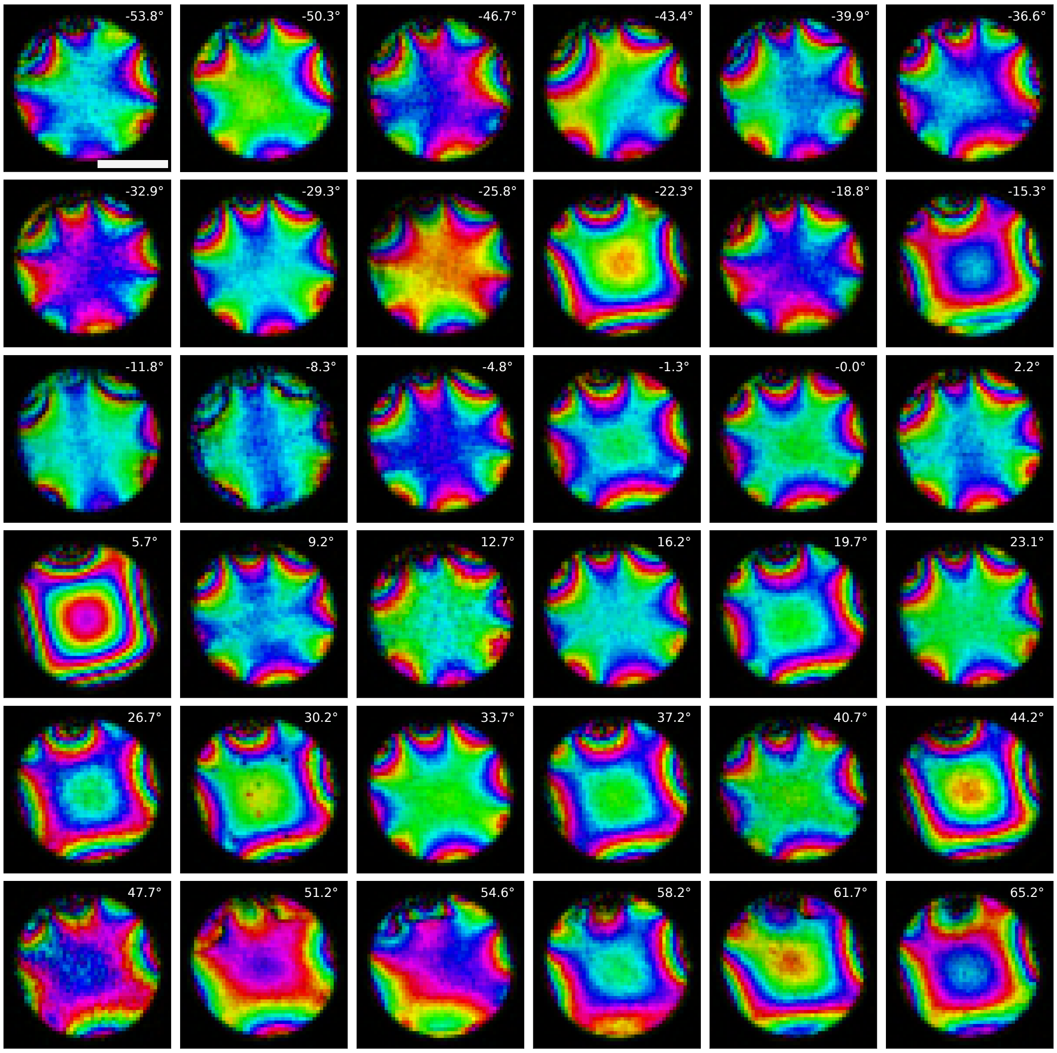

Figure 12. Fourier-space complex images of the reconstructed 1st probe mode. Scale bar: 25 mrad

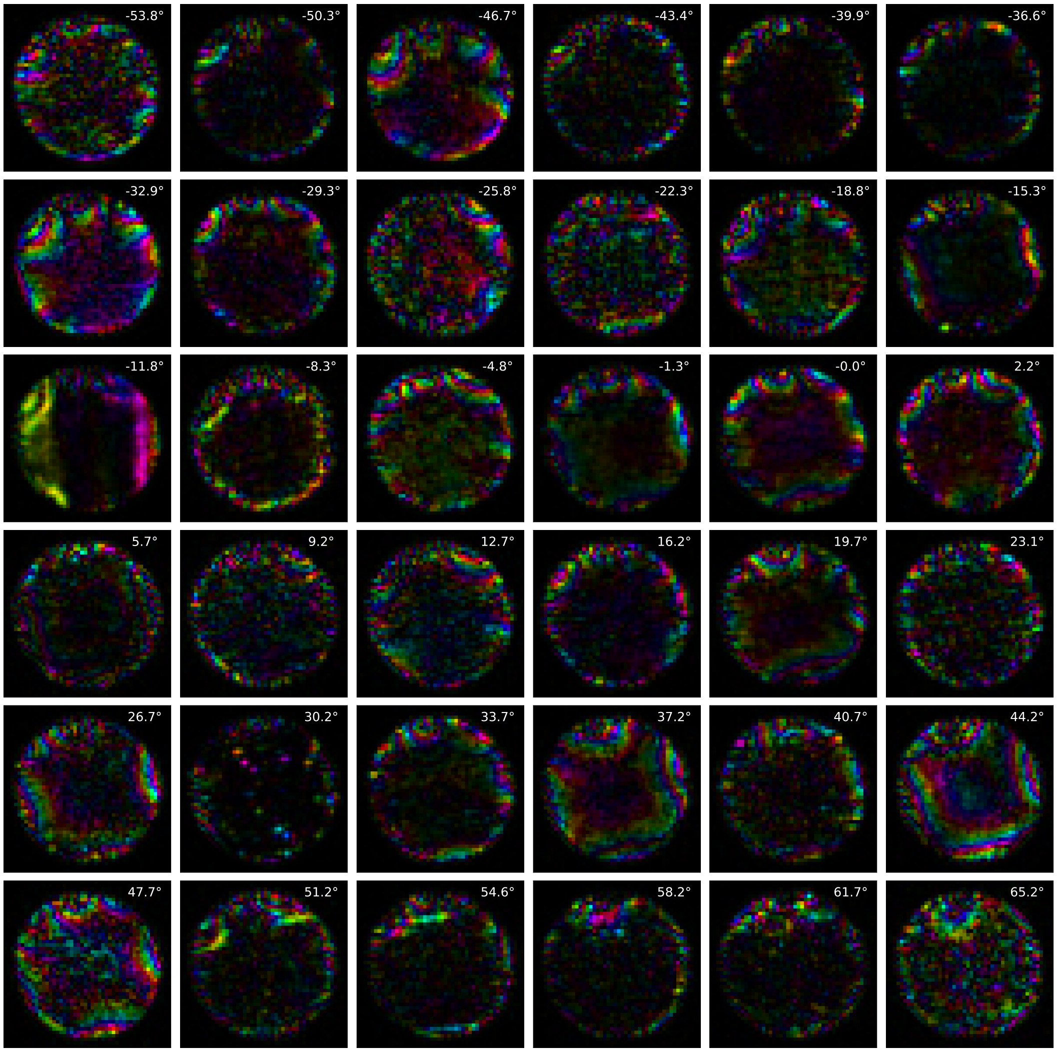

Figure 13. Fourier-space complex images of the reconstructed 2nd probe mode. Scale as in Fig. S12

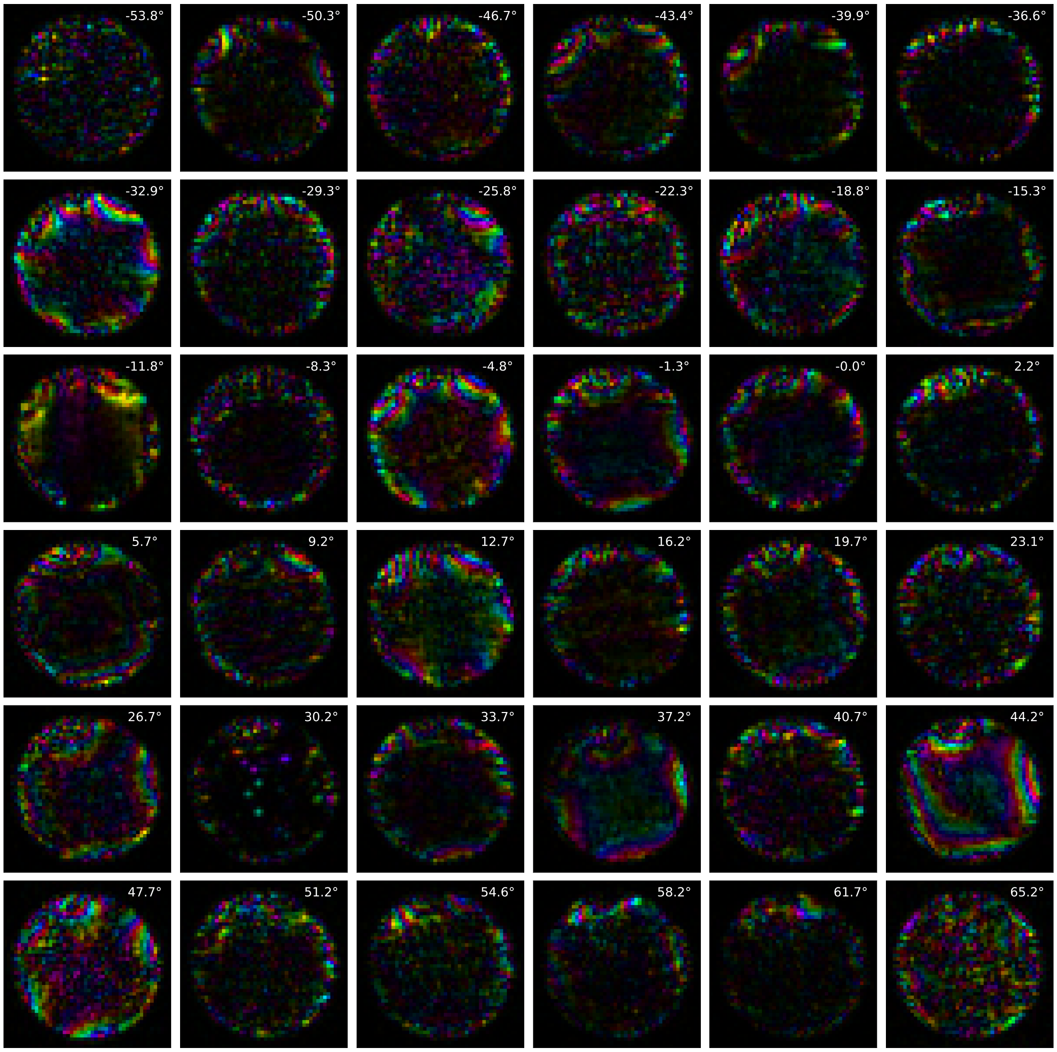

Figure 14. Fourier-space complex images of the reconstructed 3rd probe mode. Scale as in Fig. S12

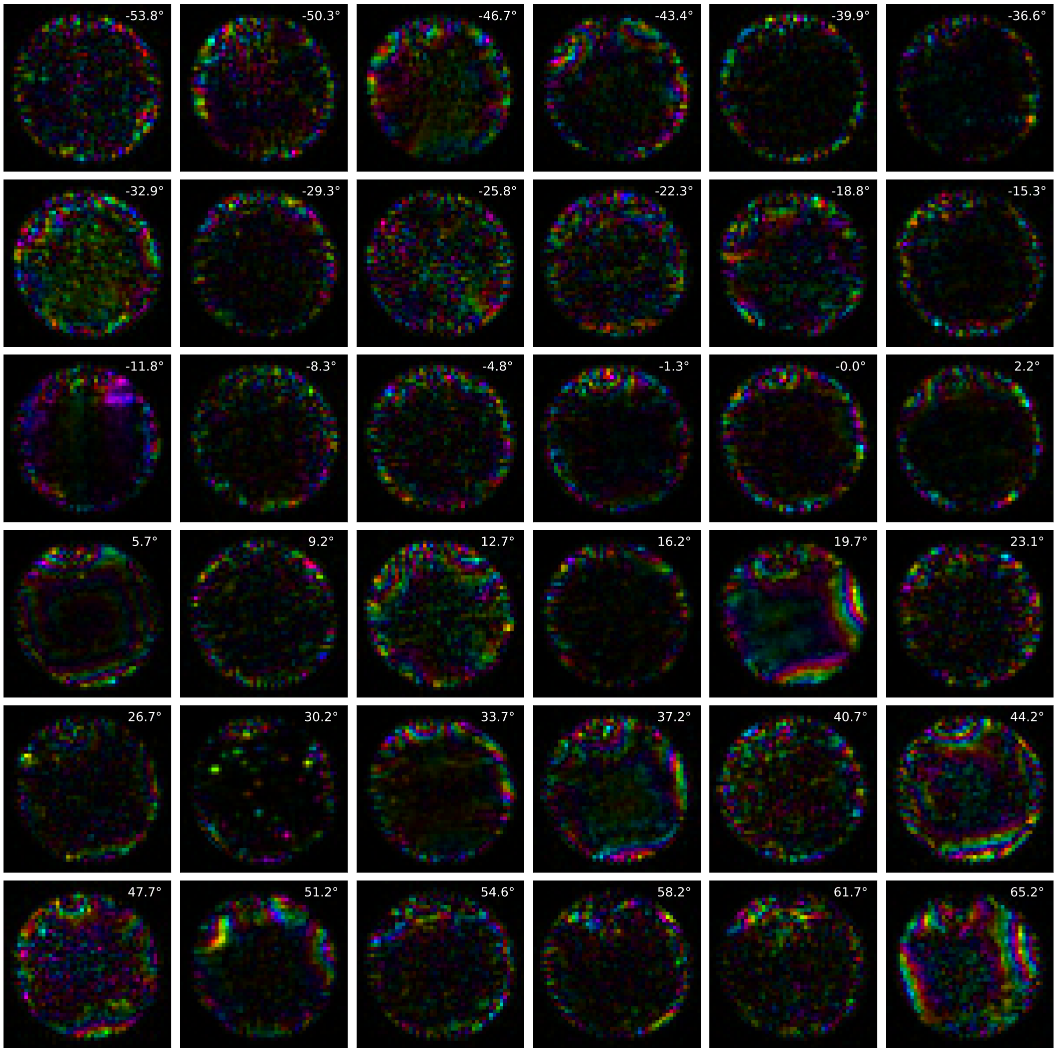

Figure 15. Fourier-space complex images of the reconstructed 4th probe mode. Scale as in Fig. S12

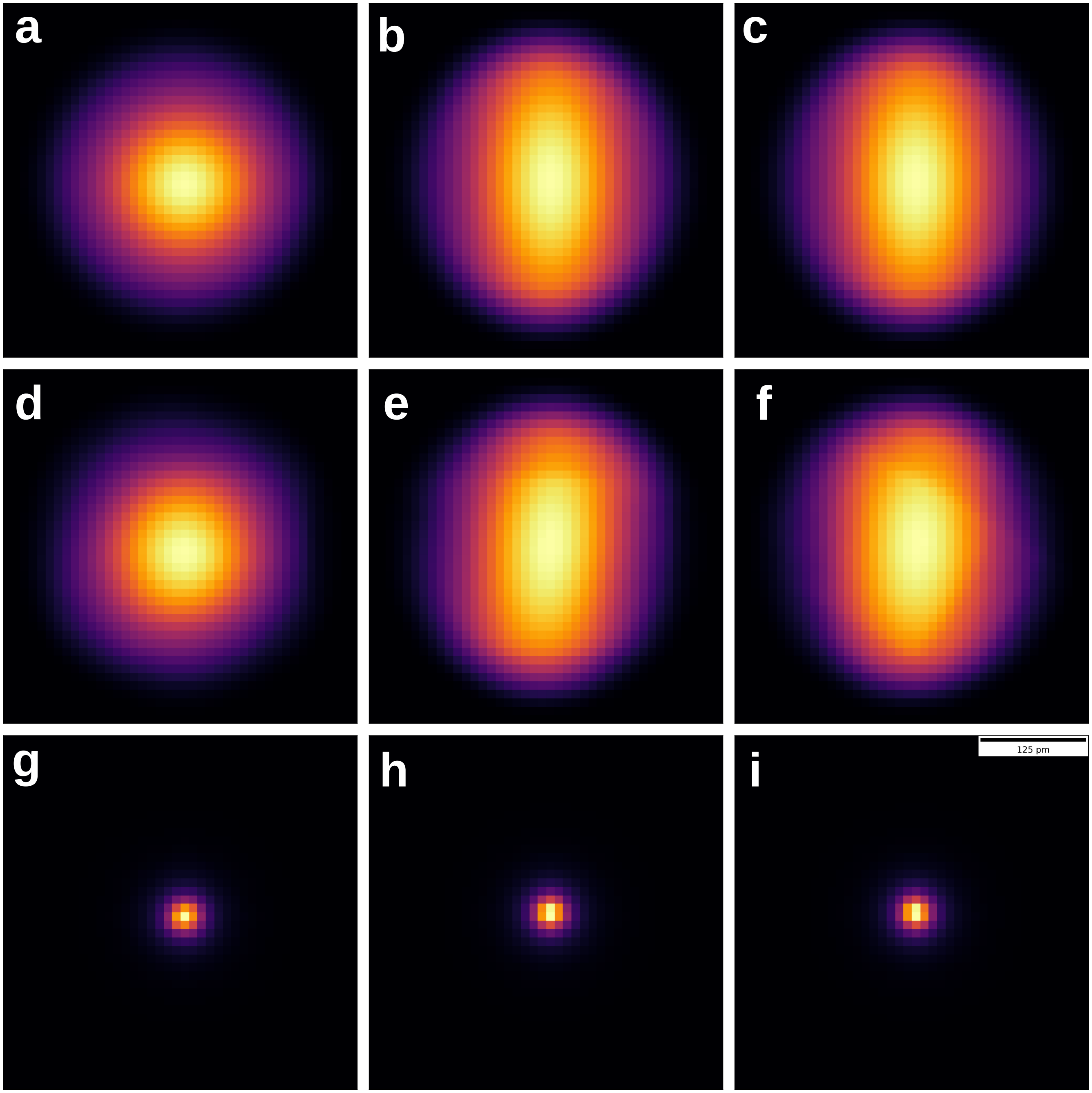

Figure 16. Left column: central slice through the Zr atomic volume, perpendicular to the missing wedge (X-Y plane). Middle column: central slice through the atomic volume (X-Z plane). Right column: central slice through the atomic volume (Y-Z plane). a)-c) Simulated potential in g)-i), convolved with the error-minimizing Gaussian. d)-f) Mean experimental Zr atom. g)-i) Simulated potential, with Debye-Waller blur from 250 Frozen phonons.

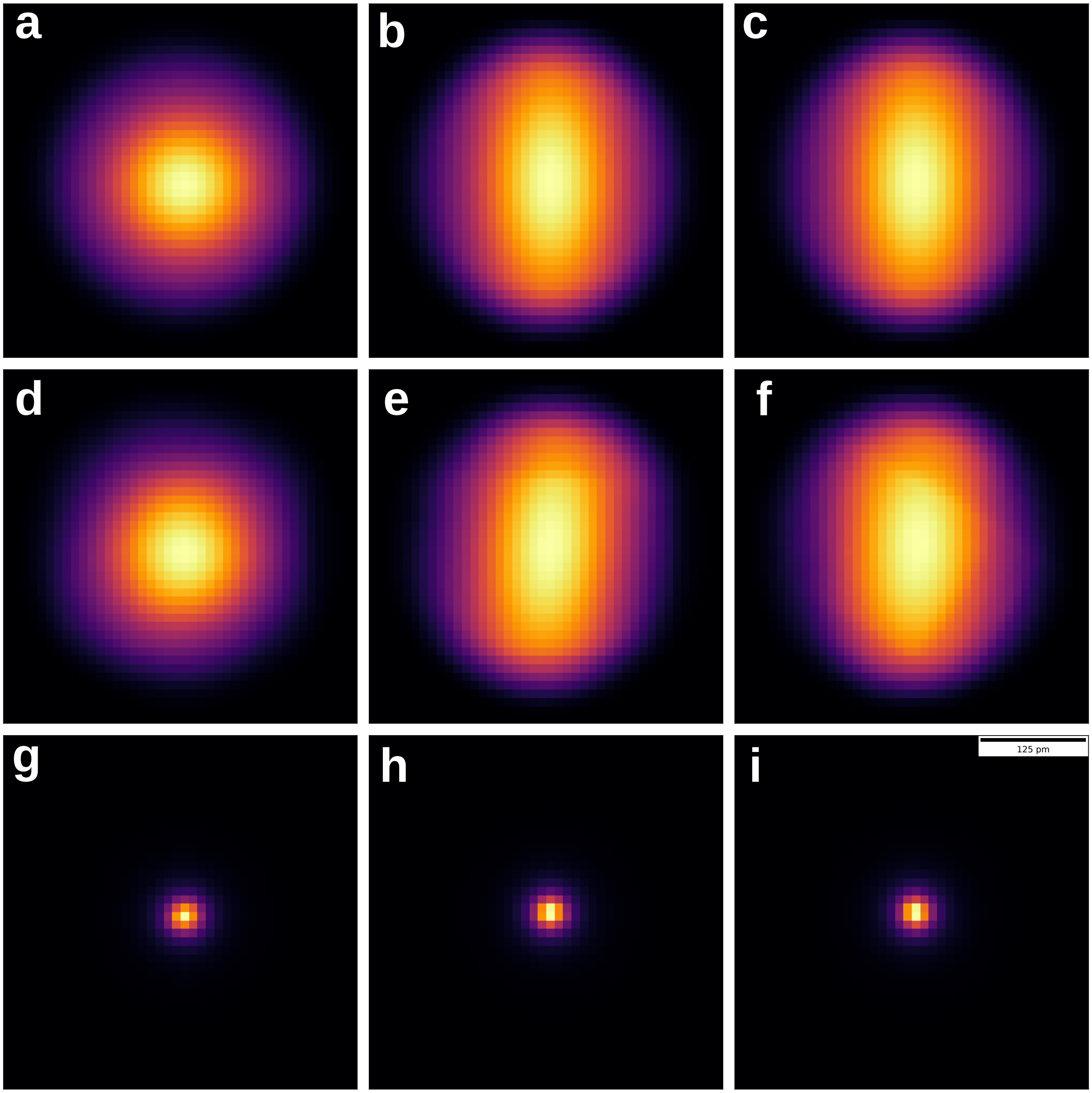

Figure 17. Left column: central slice through the Te atomic volume, perpendicular to the missing wedge (X-Y plane). Middle column: central slice through the atomic volume (X-Z plane). Right column: central slice through the atomic volume (Y-Z plane). a)-c) Simulated potential in g)-i), convolved with the error-minimizing Gaussian. d)-f) Mean experimental Zr atom. g)-i) Simulated potential, with Debye-Waller blur from 250 Frozen phonons.

DENSITY FUNCTIONAL THEORY CALCULATION DETAILS

All DFT calculations were carried out with the Vienna *Ab initio* Simulation Package (VASP) [2–4] using projector augmented wave (PAW) pseudopotentials [5, 6]. Zr(4s, 4p, 5s, 4d) and Te(5s, 5p); electrons were treated as valence , and the wave functions of the system were expanded in plane waves to an energy cutoff of 600 eV. Gamma-centered k-point grids of $14 \times 14 \times 4$, $12 \times 12 \times 1$, and $12 \times 12 \times 1$ were used for Brillouin Zone sampling of the bulk $ZrTe_5$, innercore Zr-Te tube, and central Zr-Te structure respectively. Dispersive corrections were accounted for using the optB86b-vdW of Klimeš et al. [7] which gave calculated lattice parameters for bulk $ZrTe_5$ in the *Cmcm* space group that are very close to those measured at 10 K [8]. Spin-orbit coupling was not included in the structural optimizations as it was found to have very little effect on the structural parameters for bulk $ZrTe_5$, however it was included self-consistently in the electronic structure calculations. The electronic convergence criteria set to 10^{-7} eV and the force convergence criteria set to 0.002 eV / Å.

Topological characterization using symmetry indicators and DFT calculated band structures was carried out using SYMTOPO [9]. Previous work on $ZrTe_5$ discussed how the choice of exchange-correlational functional is essential for the accurate description of its structure, and hence electronic and topological properties [8, 10, 11]. We find that the optB86b-vdW of Klimeš et al. [7] gives lattice parameters extremely close to those measured in experiment [8].

ON THE POSSIBILITY OF DETERMINING THE CHEMISTRY OF THE TE-CHAIN WITH ADF-STEM

Using multi-slice image simulations, we consider the possibility of determining the chemistry of the Te chain by tilting the sample such that the Te chain is on the edge of the nanotube and shadowed by the smallest possible number of Zr or Te atoms. We consider the case where the 30-fold dose of a single projection as used in the tomographic experiment is now used to image the perfectly oriented sample without pre-exposure, with aberration-free imaging conditions and experimental parameters as in Table I. In the simulations, we respectively swap out the Te atoms in the Te chain with Zr atoms and perform two sets of simulations with 35 noise instantiations each. The mean of the 35 images is shown in Fig 18 b) and c), while in b) the Te atoms in the Te chain were swapped with Zr atoms. 18 a) shows the orientation of the model such that the Te chains are on the outer edge. We determine the mean and standard deviation of the pixels comprising the Te and Zr atoms. These are shown in Fig. 19. It can be seen that under these perfect experimental conditions, the Zr intensity error bar is 1.58 σ from the Te mean, i.e. even under perfect conditions the decision between Te and Zr atom can be made only with less than 90 % confidence.
Real conditions in the microscope will decrease this number further. These conditions include: inability to align the nanotube to a perfect orientation without significant pre-exposure and therefore shadowing by other Zr and Te atoms, imperfect ADF-STEM detectors with Poisson-Gaussian noise, which was not modeled here, imperfect focusing, imperfect aberrations without significant pre-exposure.
We conclude that ADF-STEM experiments to determine the chemistry of the Te-chain are unlikely to yield further insights.

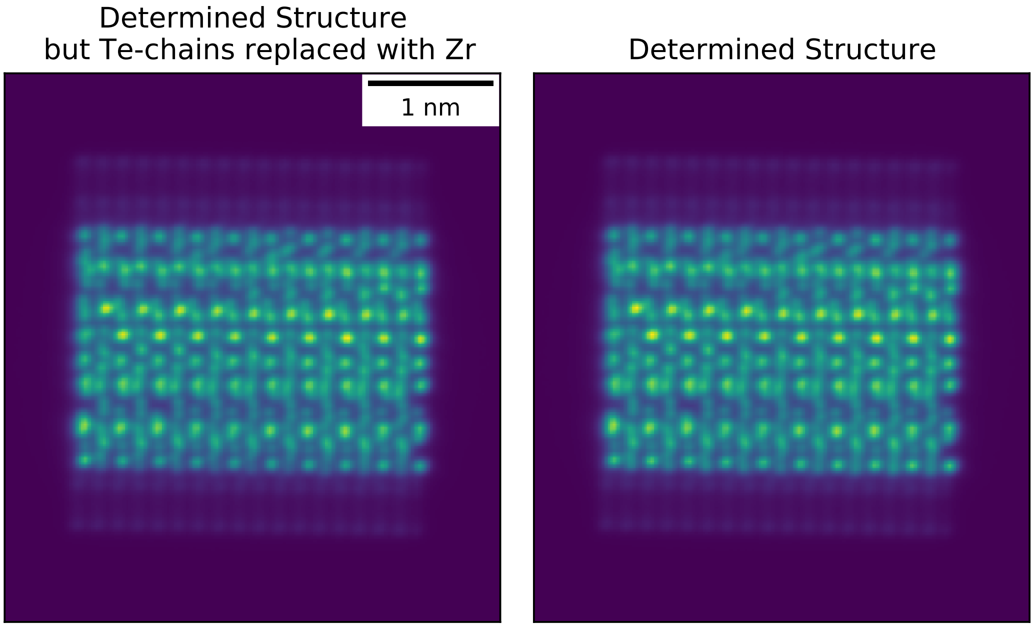

Figure 18. ADF-STEM intensity of an experiment where the nanotube is rotated such that the Te chain is at the edge of the nanotube filling and shadowed by very few other Zr or Te atoms, with a fluence of $30{*}1.72 \times 10^4\ e/\text{Å}^2$

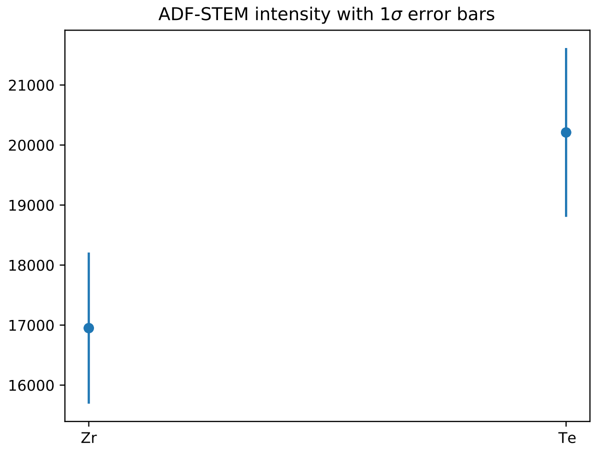

Figure 19. ADF-STEM intensity of an experiment where the nanotube is rotated such that the Te chain is at the edge of the nanotube filling and shadowed by very few other Zr or Te atoms, with a fluence of $30{*}1.72 \times 10^4\ e/\text{Å}^2$

* philipp.pelz@fau.de
† cophus@gmail.com
[1] P. M. Pelz, I. Johnson, C. Ophus, P. Ercius, and M. C. Scott, Microscopy and Microanalysis **27**, 188–189 (2021).

[2] G. Kresse and J. Hafner, Physical Review B **47**, 558 (1993).
[3] G. Kresse and J. Hafner, Physical Review B **49**, 14251 (1994).
[4] G. Kresse and J. Furthmüller, Computational Materials Science **6**, 15 (1996).
[5] P. Blöchl, Physical Review B **50**, 17953 (1994).
[6] G. Kresse and D. Joubert, Physical Review B **59**, 1758 (1999).
[7] J. c. v. Klimeš, D. R. Bowler, and A. Michaelides, Phys. Rev. B **83**, 195131 (2011).
[8] H. Fjellvåg and A. Kjekshus, Solid State Communications **60**, 91 (1986).
[9] Y. He, Y. Jiang, T. Zhang, H. Huang, C. Fang, and Z. Jin, Chinese Physics B **28**, 087102 (2019).
[10] N. L. Nair, P. T. Dumitrescu, S. Channa, S. M. Griffin, J. B. Neaton, A. C. Potter, and J. G. Analytis, Phys. Rev. B **97**, 041111 (2018).
[11] B. Monserrat and A. Narayan, Phys. Rev. Research **1**, 033181 (2019).
