# Supplementary material for: Solving complex nanostructures with ptychographic atomic electron tomography
Source: Nat Commun. 2023 Nov 30;14:7906. doi: 10.1038/s41467-023-43634-z (PMC10689721; doi:10.1038/s41467-023-43634-z)
Supplement: Supplementary file 3 — Description of Additional Supplementary Files [file 41467_2023_43634_MOESM3_ESM.pdf]

Description of Additional Supplementary Files:

Supplementary Movie 1: Video showing the 3D volume reconstruction of first the full model, and then the ZrTe nanowire. Next, both the measured atomic coordinates and volume reconstruction of each structural unit in the ZrTe nanowire are shown. Inset schematic at the top shows the position of each structural unit.
